# Self-reported vaccination-related behavior patterns among healthcare workers and the association with self-directed learning frequency: A nationwide cross-sectional survey

**DOI:** 10.3389/fpubh.2022.951818

**Published:** 2022-10-21

**Authors:** Yuan Ma, Xuan Han, Wei Li, Yuan Yang, Yunshao Xu, Di Liu, Weizhong Yang, Luzhao Feng, Libing Ma

**Affiliations:** ^1^School of Population Medicine and Public Health, Chinese Academy of Medical Sciences and Peking Union Medical College, Beijing, China; ^2^Center for Applied Statistics and School of Statistics, Renmin University of China, Beijing, China; ^3^“Breath Circles” Network Platform, Beijing, China; ^4^Department of Respiratory and Critical Care Medicine, The Affiliated Hospital of Guilin Medical University, Guilin, China

**Keywords:** self-directed learning, healthcare worker, vaccination, recommendation, public health, population medicine

## Abstract

**Background:**

Healthcare workers play an essential role in improving the public's vaccination uptake, but the full picture of such workers' engagement in vaccination-related behaviors has not been appropriately identified. According to the Integrated Theory of Health Behavior Change, self-directed learning may be a promising intervention for fostering engagement in vaccination-related behaviors, but the association between self-directed learning and such behaviors remains unclear. This study aimed to determine Chinese healthcare workers' level of engagement in behaviors for combatting vaccine-preventable diseases and assess the association between frequency of performing vaccine-focused SDL and engagement in vaccination-related behaviors.

**Materials and methods:**

An online cross-sectional survey was conducted from January 27 to February 21, 2022, using the survey platform “wjx.” Respondents were restricted to healthcare workers aged 18–65 years. A Sankey diagram and bar plots were constructed to determine patterns of engagement in a vaccination-related-behavior chain. Unconditional binary logistic regression models were fitted to determine the association between frequency of performing vaccine-focused self-directed learning and engagement in vaccination-related behaviors.

**Results:**

Of the 2,248 survey respondents, data for 2,065 were analyzed. Participants who had received influenza or pneumococcal vaccination, routinely recommended vaccination to patients, tracked patients' vaccination status, and recommended efficiently accounted for 43.2%, 50.8%, 40.3%, and 36.4% of the total participants, respectively. When only considering those who routinely made such recommendations, the proportion of those who performed tracking and efficient recommendation was 28.8% and 26.2%, respectively. When compared to performing self-directed learning “never to less than once/six months,” performing self-directed learning “more than once/week” was positively associated with being vaccinated (OR, 95% CI: 2.30, 1.74–3.03), routinely recommending vaccination (OR, 95% CI: 4.46, 3.30–6.04), and tracking the status of patients so recommended (OR, 95% CI: 6.18, 4.35–8.76).

**Conclusions:**

Chinese healthcare workers' pattern of engagement in vaccination-related behaviors must be improved. Higher frequencies of engagement in self-directed learning are associated with more active engagement in vaccination-related behaviors, meaning raising such frequencies could be a promising intervention for fostering behavior changes in this regard and ultimately increasing vaccination coverage.

## Introduction

In recent years, population medicine has become increasingly popular, which promotes a transition in health systems from treating illness to preventing illness (1, 2). Population medicine encourages healthcare workers (HCWs) to strengthen their awareness of public health and disease-prevention (1, 3) and, thus, causes them to become both beneficiaries (4) and counseling service providers in regard to vaccination uptake (5). Influenza and pneumococcal vaccines are two major contributors for the prevention of lower respiratory tract infection, which is one of the leading causes of deaths and DALYs worldwide (6). Vaccinations have been strongly recommended by the World Health Organization (7) especially for high-risk population, but the vaccination uptake still needs to be improved in some countries (8). HCWs are considered as a priority population for influenza vaccination, not only for protecting themselves but also avoiding spreading the disease to patients (9). They are also encouraged to recommend vaccination to their patients because their recommendations have a strong influence on the vaccination decisions of the general population (10). In addition, studies have indicated that HCWs' behaviors of getting vaccinated are associated with greater willingness to recommend vaccination to their patients (9). Therefore, vaccination-related behaviors among HCWs could influence the vaccination coverage in both HCWs and the public.

Previous studies have investigated receiving and recommending vaccinations among HCWs separately (11–13), but these vaccination-related behaviors could be correlated (9). For the recommendation behavior, it can be an interactive loop that HCWs might follow up the vaccination status of patients after recommending vaccinations to them, then assess the recommendation effect and continue to improve their recommendation skills according to feedback from patients, which is similar to PDCA cycle (Plan-Do-Check-Act), a scientific method to solve problems and improve continuously (14). Though the efficiency of recommendation could be a response from patients, we consider it as HCWs' behavior because it is an indispensable “check” process in the recommendation loop and also an assessment of HCWs' contributions to the vaccination uptake among the general population. Therefore, the vaccination-related-behavior chain involves receiving vaccinations, regularly recommending vaccinations to patients, tracking the vaccination status of patients who have been recommended to obtain a vaccine, and determining the efficiency of one's recommendations. All these behaviors are linked together and could reflect the full picture of the engagement of healthcare workers in vaccination. So far, the full picture of engagement in each element of the vaccination-related-behavior chain has not been appropriately identified among HCWs in China. Considering the low vaccination coverage and the essential role of HCWs in improving this situation, it is necessary to understand the current status of vaccination-related behavior patterns among HCWs and to explore associated factors.

At present, training programs are commonly used interventions to increase awareness and knowledge about vaccination among HCWs (5, 15). However, it may not be a long-term solution because HCWs usually have heavy jobs. Frequent trainings about updated vaccination knowledge would add to their burden and occupy a lot of health resources. More flexible and sustainable interventions are needed to supplement or replace this training method. According to the Integrated Theory of Health Behavior Change, health-behavior change can be enhanced by improving knowledge and beliefs, increasing self-regulation skills and abilities, and enhancing social facilitation (16). To improve HCWs' vaccine uptake and rate of recommendation of vaccines to patients, it would be insufficient to merely develop a training program that focuses only on enhancing HCWs' knowledge and beliefs about vaccination (although such efforts have previously been reported to increase engagement in vaccination behaviors) (5, 15). Person-centered interventions are needed to achieve all three components of the Integrated Theory of Health Behavior Change (16) and, notably, such interventions have previously been found to be more effective than standardized interventions for enhancing health-behavior change (17, 18). Self-directed learning (SDL) is a typical person-centered intervention, and is defined as a process by which individuals take the initiative, with or without the assistance of others, in diagnosing their learning needs, formulating learning goals, identifying human and material resources to assist their learning, choosing and implementing appropriate learning strategies, and evaluating learning outcomes (19). It is also considered a promising lifelong learning approach in the medicine field (20). Therefore, vaccine-focused SDL could be a more effective and sustainable means of fostering greater engagement in vaccination-related behaviors, but few studies have evaluated the association between SDL and such engagement.

Therefore, to obtain a comprehensive understanding of HCWs' role in combatting vaccine-preventable diseases in China and evaluate the association of vaccine-focused SDL with it, this study aimed to (1) investigate the characteristics of Chinese HCWs' engagement in the vaccination-related-behavior chain, and (2) assess the association between frequency of performing vaccine-focused SDL and engagement in vaccination-related behaviors.

## Materials and methods

### Study design

For this study, an online cross-sectional survey was jointly designed and conducted by the School of Population Medicine and Public Health, the Chinese Academy of Medical Science & Peking Union Medical College, and the “Breath Circles” platform. The “Breath Circles” platform is a media platform for HCWs with 235,000 subscribers in mainland China. The survey was published using the online survey platform “wjx” (https://www.wjx.cn) on January 27, 2022, with a link to the questionnaire being posted on the “Breath Circles” platform. Data collection finished on February 21, 2022, as no further responses were submitted after this date. This study protocol and questionnaire are approved by the Medical Ethics Committee of the Chinese Academy of Medical Sciences and Pecking Union Medical College, Beijing, China (CAMS&PUMC-IEC-2022-019). All participants had provided informed consent forms to be interviewed before logging in to fill out the questionnaire.

The survey comprised three sections: (1) Sociodemographic information (age, sex, education, years of professional experience, etc.,); (2) Vaccination-related knowledge, beliefs, and recommendation behaviors (frequency of performing vaccine-focused SDL, topics of interest, approaches used to acquire knowledge, frequency of recommending vaccination to others, etc.,); (3) Vaccination against respiratory infectious diseases (whether respondents had received influenza/pneumococcal vaccines, etc.,). We focused on vaccines related to respiratory infectious diseases (influenza and pneumococcal vaccines) when asking HCWs about their behavior of receiving vaccinations because (1) HCWs who work in healthcare settings are more likely to have respiratory infections or transmit infection to their patients; (2) HCWs are considered to be a target group for seasonal influenza vaccination by WHO (7), and those who work in hospitals are provided free influenza vaccinations in China (21). However, we did not limit types of vaccines when involving behaviors related to recommending vaccinations because HCWs from non-respiratory departments or non-hospital institutions may prefer to recommend other vaccines, such as human papillomavirus (HPV) vaccine, haemophilus influenza type b (Hib) vaccine, etc. The inclusion criterion for participants was being a HCW aged 18–65 years.

The surveyed provinces were divided into three geographic regions (eastern, central, and western regions) to reflect the regional economic development, according to the National Bureau of Statistics of China (22). Eastern regions have higher economic level and include Beijing, Tianjin, Hebei, Liaoning, Shanghai, Jiangsu, Zhejiang, Fujian, Shandong, Guangdong, and Hainan. Central regions include Shanxi, Jilin, Heilongjiang, Anhui, Jiangxi, Henan, Hubei, and Hunan. Western regions include less developed provinces (autonomous regions, municipalities): Inner Mongolia, Guangxi, Chongqing, Sichuan, Guizhou, Yunnan, Shaanxi, Gansu, and Qinghai.

### Outcome measures

The vaccination-related-behavior chain was defined as receiving vaccinations, regularly recommending vaccinations to patients, tracking the vaccination status of patients who have been recommended to obtain a vaccine, and determining the efficiency of one's recommendations (i.e., the ratio of patients who received a vaccine after being recommended to do so). The four elements were selected as indicators to evaluate the level of engagement in vaccination-related behaviors, and the latter three behaviors related to recommendation were considered sequential. However, receiving vaccinations and recommending vaccinations were not necessarily in order because it is reasonable that some HCWs recommend vaccines regularly to others without receiving influenza or pneumococcal vaccines themselves, though studies have suggested that vaccination uptake could be a driving factor in recommending vaccinations (9).

Frequency of performing vaccine-focused SDL was categorized as follows: “at least once a day,” “at least once a week,” “at least once a month,” “at least once every 6 months,” “at least once a year,” and “never”. Considering the relatively small number of people in each group, we combined these frequencies into four groups: “more than once/week,” “once/month to less than once/week,” “once/six months to once/month,” and “never to once/six months” respectively. Participants were classified as having received the influenza vaccine if they had been vaccinated for influenza during any of the latest two flu seasons (this was to allow for the potential influence of the Coronavirus Disease 2019 [COVID-19] Pandemic on respondents' ability to receive vaccinations for other diseases). Frequency of recommending vaccines to others was categorized as follows: “routinely,” “occasionally,” and “never”. The latter two groups were combined into the single group “not routinely” because of the limited sample size for these two groups. The efficiency of recommendations was categorized as follows: “all were vaccinated,” “most were vaccinated,” “a small number were vaccinated,” “none were vaccinated,” and “unclear”. In the subsequent analysis, respondents who reported that all or most of their patients received vaccinations were classified into the group “efficient recommendation,” while the remaining respondents were classified into the group “inefficient recommendation”. Those who responded “unclear” were excluded.

### Statistical analysis

The participants' basic characteristics were described in terms of their frequencies of performing vaccine-focused SDL. Continuous variables and categorical variables were represented using means ± standard deviations (SDs) [or medians (25^th^-75^th^ percentile)] and counts (percentage), respectively. Analysis of variance (or nonparametric tests) and chi-squared tests were used to examine the differences across groups. The Nightingale Rose Chart was used to categorize the learning topics and sources of those with different SDL frequencies. A Sankey diagram was created to show the flow of participants with different SDL frequencies along the behavior chain. Additionally, to explicitly compare patterns of engagement in vaccination-related behaviors among participants with different characteristics, bar plots were constructed.

To investigate the association between frequency of performing vaccine-focused SDL and engagement in vaccination-related behaviors, unconditional binary logistic regression models were used. In these models, frequency of performing vaccine-focused SDL was set as the independent variable, and being vaccinated (influenza or pneumococcal), routinely recommending vaccination, tracking the vaccination status of patients recommended to receive a vaccine, and recommendation efficiency were set as dependent variables. Odds ratios (ORs) and 95% confidence intervals (CIs) were calculated, and the group with the lowest frequency of performing vaccine-focused SDL was set as the reference group. Linear trend tests were also performed by modeling the ordered categories of vaccine-focused SDL frequency as a continuous variable in multivariate models, with the Wald test for hypothesis testing (23, 24). To check the robustness of our results, logistic regression models were also fitted for participants who work in hospitals.

Data analyses were performed using SAS (version 9.4; SAS Institute Inc.), R (version 4.1.3; R Core team 2022), and RStudio (version 2022.2.1.461; RStudio Team, 2022), applying the “ggplot2” and “eulerr” packages. All statistical tests were two-sided, with *p* < 0.05 being considered to represent statistical significance.

## Results

### Sociodemographic characteristics of the study population

Overall, 2,248 questionnaires were returned. After excluding respondents who were younger or older than the target age (18–65 years) and those who provided illogical answers, there remained 2,065 questionnaires for analysis. This sample covered 162 cities across 28 provinces (autonomous regions, municipalities) of mainland China. Most respondents were from western regions (64.21%), those from eastern regions and central regions accounted for 15.35% and 20.44%, respectively.

The characteristics of the participants, categorized in terms of their frequency of performing vaccine-focused SDL are shown in Table 1. The average age of the sample was 36.88 ± 9.35 years, and the median years of professional experience were 12.00 (5.00–20.00). Overall, 1,701 (82.4%) participants worked in hospitals, of whom 11.4, 22.7, and 66.0% worked in primary, secondary, and tertiary hospitals, respectively. The remaining 364 participants worked in other institutions, including community health centers (7.6%), Centers for Disease Control, and Prevention (3.3%), medical colleges or research institutes (4.7%), and “others” (2.3%). The participants who performed vaccine-focused SDL more frequently tended to be younger, have less professional experience, have lower educational attainment, work in lower-level hospitals, and engage in vaccination-related work.

**Table 1:** Characteristics of participants by vaccine-focused SDL frequency.

		**Vaccine-focused SDL frequency**				
	**Total** **(*N* = 2,065)**	**≥1 time/week** **(*N* = 596)**	**1 time/month to <1 time/week** **(*N* = 634)**	**1 time/6 months to <1 time/month** **(*N* = 340)**	**Never to <1 time/6 months** **(*N* = 495)**	***p***
**Age (years), Mean** **±SD**	36.88 ± 9.35	36.32 ± 9.94	37.89 ± 8.82	38.06 ± 8.64	35.47 ± 9.55	<0.001
**Age group (years)**, ***n*** **(%)**						<0.001
<30	467 (22.62)	158 (26.51)	109 (17.19)	54 (15.88)	146 (29.49)	
30–39	786 (38.06)	213 (35.74)	255 (40.22)	134 (39.41)	184 (37.17)	
40–49	592 (28.67)	162 (27.18)	201 (31.70)	111 (32.65)	118 (23.84)	
≥50	220 (10.65)	63 (10.57)	69 (10.88)	41 (12.06)	47 (9.49)	
**Sex**, ***n*** **(%)**						0.125
Male	691 (33.46)	195 (32.72)	197 (31.07)	131 (38.53)	168 (33.94)	
Female	1374 (66.54)	401 (67.28)	437 (68.93)	209 (61.47)	327 (66.06)	
**Professional experience years**, **Median (Q1–Q3)**	12.00 (5.00–20.00)	10.00 (5.00–20.00)	12.00 (7.00–20.00)	13.00 (7.00–21.00)	10.00 (4.00–17.00)	<0.001
**Professional experience years**, ***n*** **(%)**						<0.001
<5	419 (20.47)	132 (22.34)	93 (14.86)	58 (17.11)	136 (27.70)	
5–9	379 (18.51)	126 (21.32)	104 (16.61)	54 (15.93)	95 (19.35)	
10–14	428 (20.91)	96 (16.24)	159 (25.40)	75 (22.12)	98 (19.96)	
15–19	283 (13.83)	82 (13.87)	89 (14.22)	47 (13.86)	65 (13.24)	
≥20	538 (26.28)	155 (26.23)	181 (28.91)	105 (30.97)	97 (19.76)	
**Educational attainment**						<0.001
Bachelor's degree and below	1,581 (76.56)	510 (85.57)	481 (75.87)	246 (72.35)	344 (69.49)	
Master's degree and above	484 (23.44)	86 (14.43)	153 (24.13)	94 (27.65)	151 (30.51)	
**Institution**, ***n*** **(%)**						
Hospital	1,701 (82.37)	464 (77.85)	534 (84.23)	295 (86.76)	408 (82.42)	
Community health center	157 (7.60)	61 (10.23)	54 (8.52)	17 (5.00)	25 (5.05)	
CDC	68 (3.29)	30 (5.03)	14 (2.21)	12 (3.53)	12 (2.42)	
Medical schools or research institutes	97 (4.70)	29 (4.87)	22 (3.47)	11 (3.24)	35 (7.07)	
Others	42 (2.03)	12 (2.01)	10 (1.58)	5 (1.47)	15 (3.03)	
**Occupation**, ***n*** **(%)**						<0.001
Doctor	1,111 (54.92)	284 (48.63)	368 (58.97)	197 (58.81)	262 (54.58)	
Nurse	530 (26.20)	191 (32.71)	159 (25.48)	78 (23.28)	102 (21.25)	
Technician	118 (5.83)	30 (5.14)	33 (5.29)	23 (6.87)	32 (6.67)	
Medical school students or researchers	168 (8.30)	48 (8.22)	38 (6.09)	19 (5.67)	63 (13.13)	
Others	96 (4.75)	31 (5.31)	26 (4.17)	18 (5.37)	21 (4.38)	
**Hospital level**, ***n*** **(%)**						<0.001
Primary	193 (11.35)	84 (18.10)	59 (11.05)	32 (10.85)	18 (4.41)	
Secondary	386 (22.69)	128 (27.59)	120 (22.47)	56 (18.98)	82 (20.10)	
Tertiary	1,122 (65.96)	252 (54.31)	355 (66.48)	207 (70.17)	308 (75.49)	
**Department**, ***n*** **(%)**						0.014
Respiratory	864 (50.79)	207 (44.61)	285 (53.37)	150 (50.85)	222 (54.41)	
Others	837 (49.21)	257 (55.39)	249 (46.63)	145 (49.15)	186 (45.59)	
**Job title**, ***n*** **(%)**						<0.001
Junior	524 (25.38)	196 (32.89)	150 (23.66)	69 (20.29)	109 (22.02)	
Middle	714 (34.58)	180 (30.20)	235 (37.07)	126 (37.06)	173 (34.95)	
Senior	595 (28.81)	153 (25.67)	200 (31.55)	119 (35.00)	123 (24.85)	
None	232 (11.23)	67 (11.24)	49 (7.73)	26 (7.65)	90 (18.18)	
**Work is related to vaccination**, ***n*** **(%)**	795 (38.50)	340 (57.05)	281 (44.32)	98 (28.82)	76 (15.35)	<0.001
Geographic regions^*^, ***n*** **(%)**						<0.001
Eastern regions	317 (15.35)	94 (15.77)	78 (12.30)	43 (12.65)	102 (20.61)	
Central regions	422 (20.44)	84 (14.09)	118 (18.61)	71 (20.88)	149 (30.10)	
Western regions	1,326 (64.21)	418 (70.13)	438 (69.09)	226 (66.47)	244 (49.29)	
**Vaccination-related behaviors**, ***n*** **(%)**						
Receiving vaccinations	893 (43.24)	322 (54.03)	272 (42.90)	143 (42.06)	156 (31.52)	<0.001
Recommending routinely	1049 (50.80)	405 (67.95)	350 (55.21)	155 (45.59)	139 (28.08)	<0.001
Tracking the vaccination status of people who were recommended^†^	833 (44.10)	382 (66.78)	272 (44.81)	113 (34.98)	66 (17.05)	<0.001
Recommending efficiently^††^	751 (91.36)	355 (93.67)	242 (90.30)	100 (89.29)	54 (85.71)	0.107

* Regions were divided according to the National Bureau of Statistics of China. Eastern regions include Beijing, Tianjin, Hebei, Liaoning, Shanghai, Jiangsu, Zhejiang, Fujian, Shandong, Guangdong, and Hainan. Central regions include Shanxi, Jilin, Heilongjiang, Anhui, Jiangxi, Henan, Hubei, and Hunan. Western regions include Inner Mongolia, Guangxi, Chongqing, Sichuan, Guizhou, Yunnan, Shaanxi, Gansu, and Qinghai.

† For tracking the vaccination status of people who were recommended, the denominator was the number of participants who have ever recommended patients to receive vaccinations.

†† For recommending efficiently, the denominator was the number of participants who tracked and knew the vaccination status of those who were recommended.

### Topics and sources for vaccine-focused self-directed learning

**Figure 1:**
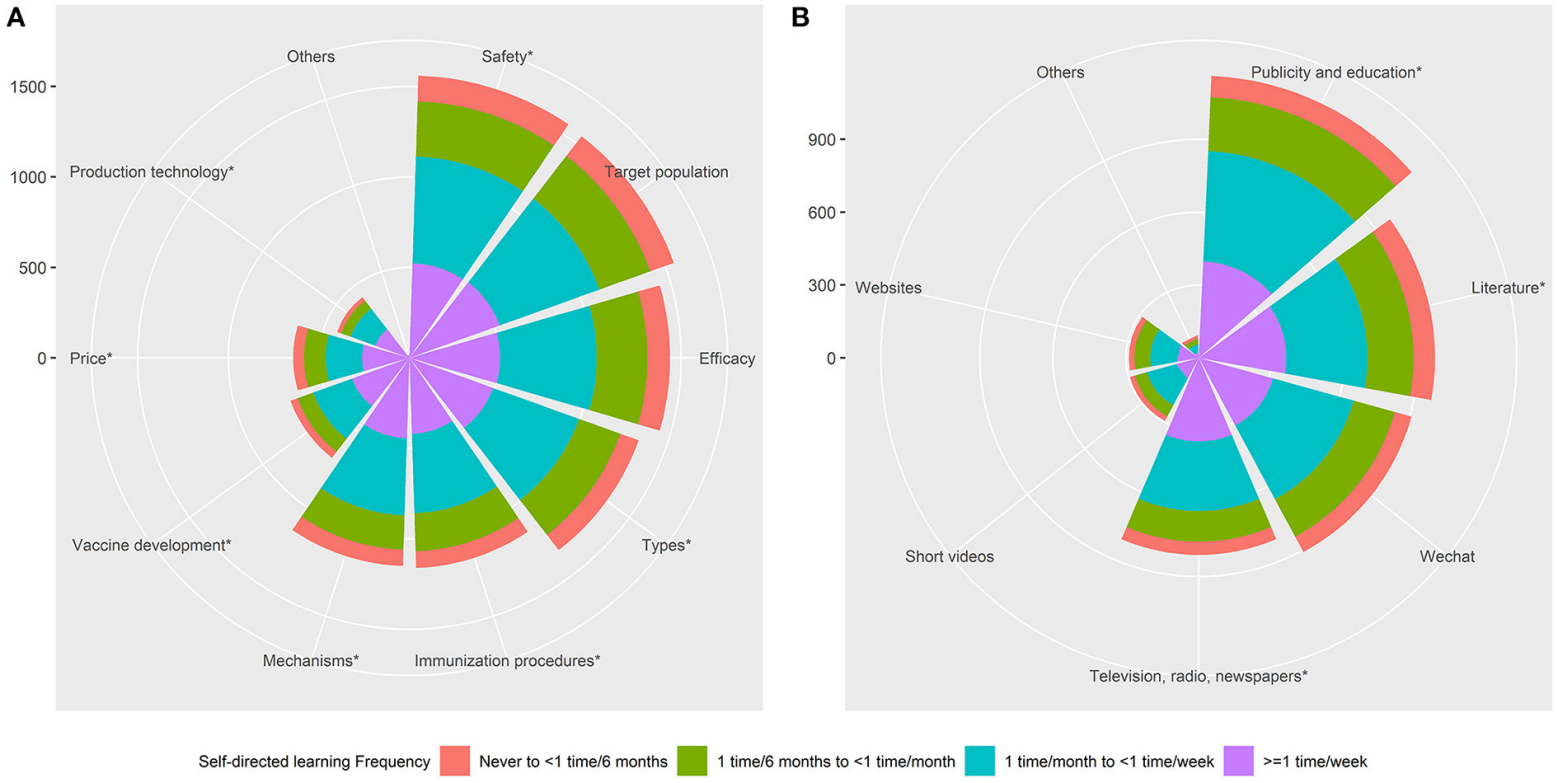
Nightingale Rose Chart for learning topics and sources in participants with different SDL frequencies. **(A)** Learning topics. **(B)** Learning sources. Note: The *symbol indicates that there is a significant difference among people with different SDL frequencies on this item.

The three main vaccine-related topics that the participants investigated in their SDL were vaccine safety (90.4%), target populations (89.8%), and vaccine efficacy (83.3%). Vaccine types (77.9%), immunization procedures (67.3%), and how vaccines function (66.6%) were also important SDL topics (Figure 1A, Supplementary Table S1). The most common source of knowledge was publicity and education efforts in communities and hospitals (67.3%), followed by books or monographs (56.3%), and WeChat (52.6%; Figure 1B, Supplementary Table S2), respectively. The learning topics and sources for participants with different SDL frequencies are shown in Figures 1A,B.

### Pattern of vaccination-related behaviors

**Figure 2:**
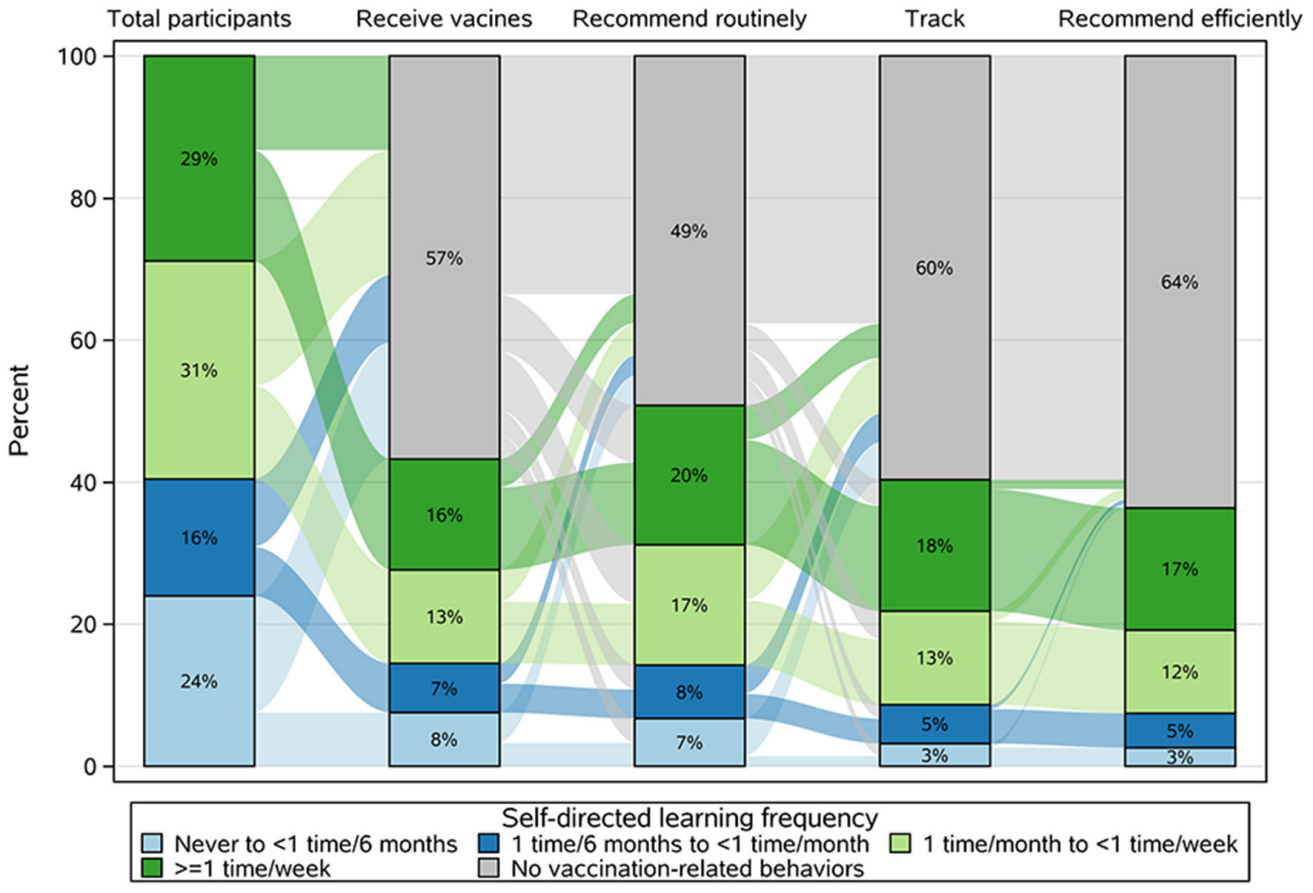
Sankey diagram showing the pattern of vaccination-related behaviors among participants with different SDL frequencies.

**Figure 3:**
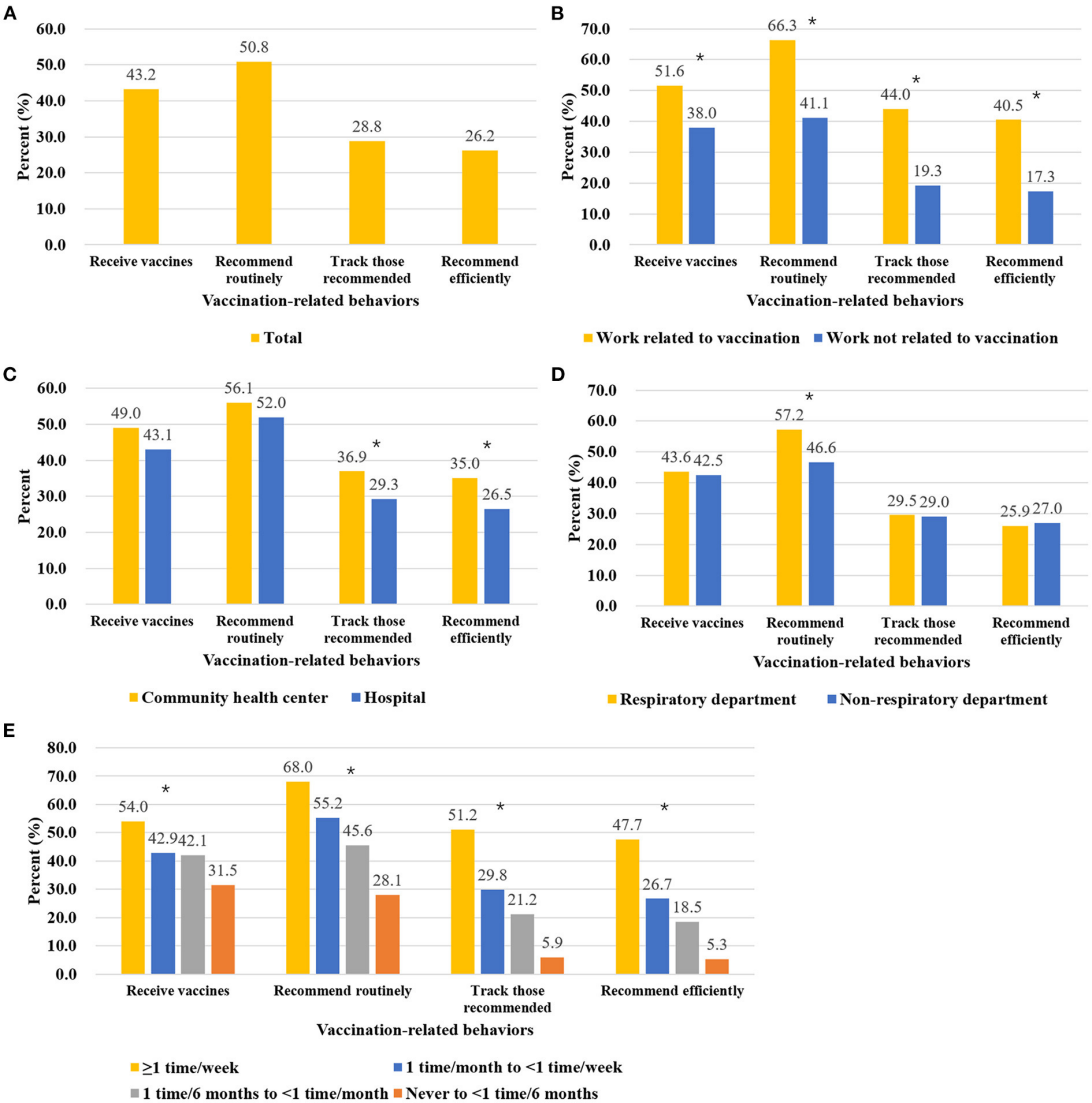
Engagement in vaccination-related behaviors among different groups of participants. **(A)** Total participants. **(B)** Participants performing vaccination-related work or not. **(C)** Participants working in hospitals or community health centers. **(D)** Participants working in respiratory or non-respiratory departments. **(E)** Participants with different SDL frequencies. When evaluating the engagement in behaviors of tracking or efficient recommendation, only participants who recommend vaccination routinely were included. Chi-square tests were applied to test the difference among different groups of participants, and the *symbol indicates p-value of <0.05.

A Sankey diagram was constructed to illustrate the flow of engagement in vaccination-related behaviors among participants with different SDL frequencies (Figure 2). This diagram showed the behavior chain for receiving vaccines, routinely recommending vaccination to others, tracking the vaccination status of those recommended to receive vaccines, and efficient recommendation. Overall, 43, 51, 40, and 36% of the total participants performed each of the above behaviors, respectively. When only considering those who performed routine recommendations, those who performed tracking and efficient recommendation accounted for 28.8% and 26.2%, respectively (Figure 3A). Influenza vaccines, COVID-19 vaccines, and pneumococcal vaccines were the leading three vaccines that participants have recommended (Supplementary Table S3). The primary reasons participants did not recommend vaccination were inadequate knowledge about vaccines or target populations and an absence of national or workplace requirements to do so (Supplementary Table S4). The transitions of participants with different SDL frequencies along the behavior chain were also displayed in the diagram (Figure 2). Among the participants with the highest SDL frequency (28.9% of the total participants), 322 (15.6% of the total participants) performed the first behavior, and 355 (17.2% of the total participants) performed the final behavior. As a contrast, the participants with the lowest SDL frequency (24.0% of the total participants) fell from 156 (7.6%) to 54 (2.6%) across these stages, respectively.

Participants who performed vaccination-related work showed much higher rates of engagement in vaccination-related behaviors (51.6% vs. 38.0% for vaccination uptake, 66.3% vs. 41.1% for routine recommendation, 44.0% vs. 19.3% for tracking, and 40.5% vs. 17.3% for efficient recommendation) (Figure 3B). Participants working in community health centers performed better than those working in hospitals for all elements of the behavior chain (49.0% vs. 43.1% for vaccination uptake, 56.1% vs. 52.0% for routine recommendation, 36.9% vs. 29.3% for tracking, and 35.0% vs. 26.5 for efficient recommendation, though only the latter two comparisons had significant differences) (Figure 3C). Among participants who worked in hospitals, the rate of engagement in routine recommendation was approximately 10% higher among those who worked in respiratory departments than those who worked in non-respiratory departments (Figure 3D). Statistical tests were provided in Supplementary Table S5.

Higher SDL frequency was associated with higher engagement in all vaccination-related behaviors (Figure 3E). For the participants with the highest SDL frequency, 54.0, 68, and 51.2% were vaccinated, routinely recommended vaccination, and tracked patients' vaccination status, respectively; meanwhile, for the participants with the lowest SDL frequency, these rates were 31.5, 28.1, and 5.9%, respectively. Finally, across the four SDL groups, of those who routinely recommended vaccination and tracking, 93.1, 89.4, 87.5, and 89.7% (from high SDL frequency to low SDL frequency, respectively) did so efficiently.

### Association between performing self-directed learning and engagement in vaccination-related behaviors

After controlling for covariates, frequency of performing vaccine-focused SDL was positively associated with engagement in all vaccination-related behaviors except efficient recommendation (Table 2). When compared with the SDL frequency of never to less than once/six months, the SDL frequency of more than once/week was associated with 2.30-times higher odds of being vaccinated, 4.46-times higher odds of routinely recommending vaccination, and 6.18-times higher odds of tracking the status of patients recommended to receive vaccination. A monotonic increase in ORs with increasing SDL frequency was observed for these three behaviors (*p*-values for trend: <0.001). Similar results were also observed for those working in hospitals (Table 2).

**Table 2:** Associations between vaccine-focused SDL frequency and vaccination-related behaviors.

	**Model 1^a^** **All participants**		**Model 2^b^** **Participants working in hospitals**	
**Vaccine-focused SDL frequency**	Case/ Control^*^	**OR (95% CI)**	**Case/ Control**	**OR (95% CI)**
**Receiving vaccinations**	893 / 1,172		733 / 968	
Never to <1 time/6 months	156 / 339	Ref	132 / 276	Ref
1 time/6 months to <1 time/month	143 / 197	1.46 (1.08–1.98)	126 / 169	1.44 (1.04–1.99)
1 time/month to <1 time/week	272 / 362	1.50 (1.15–1.96)	229 / 305	1.38 (1.03–1.85)
≥1 time/week	322 / 274	2.30 (1.74–3.03)	246 / 218	2.11 (1.55–2.88)
*P* trend		<0.001		<0.001
**Recommend routinely**	1,049 / 1,016		884 / 817	
Never to <1 time/6 months	139 / 356	Ref	124 / 284	Ref
1 time/6 months to <1 time/month	155 / 185	1.60 (1.16–2.20)	139 / 156	1.67 (1.19–2.35)
1 time/month to <1 time/week	350 / 284	2.30 (1.74–3.05)	298 / 236	2.19 (1.62–2.97)
≥1 time/week	405 / 191	4.46 (3.30–6.04)	323 / 141	4.80 (3.43–6.72)
*P* trend		<0.001		<0.001
**Track the vaccination status of those**	833 / 1,056		692 / 889	
**recommended to receive a vaccine**				
Never to <1 time/6 months	66 / 321	Ref	57 / 273	Ref
1 time/6 months to <1 time/month	113 / 210	2.16 (1.48–3.14)	91 / 193	1.80 (1.20–2.69)
1 time/month to <1 time/week	272 / 335	3.08(2.20–4.31)	231 / 283	2.92 (2.04–4.18)
≥1 time/week	382 / 190	6.18 (4.35–8.76)	313 / 140	6.33 (4.33–9.27)
*P* trend		<0.001		<0.001
**Recommend efficiently**	751 / 71		623 / 60	
Never to <1 time/6 months	54 / 9	Ref	46 / 8	Ref
1 time/6 months to <1 time/month	100 / 12	1.10 (0.40–3.00)	79 / 11	1.01 (0.34–3.01)
1 time/month to <1 time/week	242 / 26	1.35 (0.55–3.29)	205 / 23	1.27 (0.49–3.30)
≥1 time/week	355 / 24	1.99 (0.79–5.04)	293 / 18	2.28 (0.82–6.35)
*P* trend	751 / 71	0.080		0.052

a Model 1: For the model of receiving vaccinations, covariates included age (<30, 30–39, 40–49, ≥50), sex (Male, Female), years of professional experience (<5, 5–9, 10–14, 15–19, ≥20), educational attainment (Bachelor's degree and below, Master's degree and above), institution (Hospital, Community health center, CDC, Medical schools or research institutes, Others), occupation (Doctor, Nurse, Technician, Medical school students or researchers, Others), job title (Junior, Middle, Senior, None), whether perform vaccination-related work (Yes, No), and geographic regions (Eastern regions, Central regions, Western regions). For models of recommending vaccination routinely, whether getting influenza/ pneumococcal vaccination (Yes, No) was further adjusted. For models of recommending vaccination efficiently, whether getting influenza/ pneumococcal vaccination (Yes, No) and whether recommend routinely (Yes, No) were further adjusted.

b Model 2: For the model of receiving vaccinations, covariates included age (<30, 30–39, 40–49, ≥50), sex (Male, Female), years of professional experience (<5, 5–9, 10–14, 15–19, ≥20), educational attainment (Bachelor's degree and below, Master's degree and above), occupation (Doctor, Nurse, Technician, Others), department (Respiratory, non-respiratory), job title (Junior, Middle, Senior, None), whether perform vaccination-related work (Yes, No), whether one works in a hospital that offers on-site vaccination (Yes, No), and geographic regions (Eastern regions, Central regions, Western regions). For models of recommending vaccination routinely, whether getting influenza/ pneumococcal vaccination (Yes, No) was further adjusted. For models of recommending vaccination efficiently, whether getting influenza/ pneumococcal vaccination (Yes, No) and whether recommend routinely (Yes, No) were further adjusted.

* Case represents the number of participants with the indicated behavior, and Control represents the number of participants without the indicated behavior. For tracking vaccination status, the sum of Case and Control was the number of participants who have ever recommended patients to receive vaccinations. For recommending efficiently, the sum of Case and Control was the number of participants who tracked and knew the vaccination status of those who were recommended.

In addition, HCWs with lower levels of education, vaccination-related work, history of influenza or pneumococcus vaccination, doctors, and those in central or western regions of China were more likely to make vaccination recommendations. HCWs with vaccination-related work, nurses, and those in eastern regions of China were more likely to be vaccinated (Supplementary Tables S6, S7).

## Discussion

According to the concept of population medicine, to achieve improvements in population health, HCWs should have both individual- and population-based health perspectives, and incorporate disease-prevention into clinical practice (1, 2). Vaccination has been proven to be an efficient approach for disease-prevention and improving public health, and the role of HCWs in promoting vaccination has been highlighted in several previous studies (5). The present study investigated Chinese HCWs' patterns of engagement in a vaccination-related-behavior chain, and the impact that their frequency of vaccine-focused SDL has on these patterns. This chain comprised receiving vaccinations, routinely recommending vaccination to patients, tracking the vaccination status of patients so recommended, and efficiently recommending vaccination. Overall, 43, 51, 40, and 36% of the total participants of this study performed these behaviors, respectively. When only considering participants who routinely recommended vaccination, 28.8% and 26.2% tracked vaccination status and recommended efficiently, respectively. Participants whose work was related to vaccination and those who worked in community health centers or the respiratory departments of hospitals performed better in this regard. Also, higher SDL frequency was associated with greater engagement in vaccination-related behaviors.

Studies have shown that HCWs are twice as likely to be infected with influenza when compared to the general population (4), and that vaccination can reduce illness-related absences among HCWs (25). To increase the vaccination rate among HCWs, the Chinese government has mandated that hospitals provide free influenza vaccinations for such workers (21). However, the rate of influenza and pneumococcal vaccination among HCWs in hospitals remains low, as shown in this study (43.1%). This rate is higher than that reported for seasonal influenza vaccination during the 2018–2019 season (11.6%) (12), but still lower than rates in other countries, such as England (69% in the 2017–2018 season) (26) and the United States (78.4% in the 2017–2018 season) (27). The rate observed in the present study may have been impacted by the COVID-19 Pandemic, which probably disrupted HCWs' routines regarding obtaining vaccinations; however, in this study participants were considered to have received the influenza vaccine if they had gotten vaccinated during any of the previous two flu seasons.

The national influenza-prevention policy of China suggests that recommendations from HCWs are an essential means of promoting influenza vaccination (28). In this study, only half of the total participants routinely recommended vaccination, which is similar to that reported in other studies conducted in China (29, 30). Those who performed vaccination-related work showed an approximately 15%-higher recommendation rate than those who performed other work, and those from respiratory departments showed an approximately 10%-higher rate than those from other departments. Thus, the specific professions of HCWs may be associated with their engagement in vaccination-related behaviors. This accords with the findings of a study that examined professionals who had important roles regarding public human papillomavirus vaccination; the study found that such individuals engaged in relatively higher recommendation behavior (74.8%) (13). Participants who worked in community health centers showed higher recommendation willingness than those who worked in hospitals, which is consistent with previous studies (29, 31). Additionally, an absence of national or workplace requirements to provide vaccination recommendations to patients was reported as being a primary reason participants were unwilling to recommend vaccination. It suggests that it is necessary to organize programs or training to help health professionals understand vaccination policy and enhance their awareness of their roles in public vaccination.

Among participants who routinely recommended vaccination to patients, only 40% tracked the vaccination status of the patients to whom they had made such recommendations. However, the latter group showed a high efficiency of recommendation that over 90% of participants reported that most of their patients received vaccination after being recommended. It is not possible to determine the vaccination status of the untracked patients, but it is reasonable to speculate that patients who were aware that HCWs were tracking their vaccination status had higher motivation to obtain vaccination. Besides, there was no significant difference in the efficiency of recommendation among HCWs with different characteristics (e.g., knowledge, occupation) (32), which suggests that all HCWs' recommendations about vaccination have positive effect on patients' vaccination decisions. Therefore, we consider it a good intervention to encourage HCWs to recommend vaccinations to their patients. Though we did not find any association between the efficiency of recommendation and HCWs' characteristics, other factors that we did not include in the analysis may influence the recommendation efficiency, such as patients' own perceptions about diseases and vaccines, psychological and social context (e.g., support of family, National Immunization Program vaccines), and practical issues (e.g., affordability, ease of access) (33). Promoting vaccination uptake is a comprehensive issue that it might be insufficient to rely only on HCWs' recommendations. Instead, integrated interventions should be developed, including publicity and education efforts of communities, the positive leading of mass media, government financial support for vaccination.

SDL is an essential life-long learning practice for HCWs, but the current situation regarding HCWs' level of engagement in vaccine-focused SDL must be improved. In this study, almost one-quarter of the participants did not perform vaccine-focused SDL on even a semi-annual basis (although 60% reported that they did so at least once a month). Knowledge limitations have been identified as obstacles to HCWs' vaccination uptake and willingness to recommend vaccination to patients, while greater knowledge has been determined to be a key predictor of HCWs' likelihood of recommending vaccines (because it instills greater confidence regarding counseling patients about vaccines) (13, 34). As expected, in this study SDL was found to be positively associated with active engagement in vaccination behaviors. There was a decreasing trend in engagement in vaccination-related behaviors as SDL frequency decreased. When compared to those with the lowest SDL frequency, the participants with the highest SDL frequency were 2.3-times more likely to uptake vaccination, 4.5-times more likely to recommend vaccination, and 6.2-times more likely to track the vaccination status of patients so recommended. This finding may be explained by the fact that SDL is as effective as traditional teaching methods for improving health professionals' education (35), and is associated with problem-solving ability, which is necessary when offering consultation services for patients (36). Therefore, along with providing training for HCWs, which has been considered by many researchers (37, 38), promoting engagement in vaccine-focused SDL could also play an important role in improving vaccination coverage. Additionally, compared with traditional education and training, SDL, which is a person-centered intervention, involves self-regulation skills and abilities, and allows learners to transfer proximal learner outcomes into distal (long-term) outcomes (16). Hence, it can be suggested that SDL is associated with more sustainable behavior change than standard educational training. However, further investigation is needed to compare these two approaches in practice.

There are some other crucial factors associated with HCWs' engagement with the vaccination-related behavior chain. Although studies have indicated that education can have both a positive and negative effect on vaccine acceptance (39), this study showed that HCWs with higher educational attainment are more likely to receive vaccines; such experience with vaccination could, in turn, increase the likelihood that these workers routinely recommend vaccines and perform tracking of patients so recommended. This is consistent with the view that improving the vaccination uptake among HCWs could have a positive influence on public vaccination (40, 41). We also found that, among our participants, performing vaccine-related work was associated with higher rates of vaccination uptake, routine recommendation, and tracking; this could be explained by the fact that such workers were more knowledgeable about vaccines (and may have had greater recognition of their responsibility for public health in this regard) (40, 41). In China, HCWs' duties regarding vaccination or health education of the public can vary; for example, community health centers are the main institutions that provide vaccination services. Thus, HCWs who work in other facilities may be unaware of the importance of vaccine recommendation and tracking. Therefore, such workers should be provided with training to change their beliefs and attitudes toward public health and disease-prevention. It is also noticeable that on-site vaccination in hospitals increases vaccination coverage among HCWs (because of convenient access to vaccination services), but does not influence the regularity of their recommendations or their recommendation efficiency. This may be because, to get a vaccination, the general population is usually required to make an appointment with a community health center rather than receive a vaccination directly in a hospital. It would be helpful to grant HCWs the authority to provide vaccinations to patients who are willing to get vaccinated in the hospital. Regional economic level was also an associated factor that HCWs from more developed regions tended to receive vaccines while those from less developed regions were more likely to recommend vaccination to patients. The low vaccination uptake in less developed regions is consistent with previous studies (33, 42), which may be due to limited medical resources or insufficient education about vaccination in less developed regions. The reason for the more active recommending behavior in less developed regions could be that HCWs there may have fewer daily visits and have enough time to provide counseling services to their patients.

However, this study nevertheless has several limitations. The sample size was limited and the self-selection bias could exist as many other web surveys because the study let HCWs decide if they would like to participate in the survey, which may result in a sample of individuals that is not representative of the overall population. For instance, it is possible for these participants to have more exposure to self-learning materials than those non-subscribers of the media platform and our study may have overestimated the self-learning behaviors. However, the promotion of SDL is highlighted because the real gap between the current situation of SDL behavior and the expectation is even larger. For the association between SDL behavior and vaccination-related behaviors, the estimation bias could be reduced because we adjusted several sociodemographic characteristics of these participants that might confound our estimation. Besides, the HCWs examined mainly worked in the respiratory departments of hospitals, which could have led to selection bias. However, workers in hospital respiratory departments are a high-risk population for vaccine-preventable infectious diseases such as influenza and pneumococcal diseases, and their engagement in vaccination-related behaviors would be expected to be better than others. Therefore, this study, by determining their actual level of engagement in vaccination-related behaviors, could clarify the gap between reality and expectation. In addition, vulnerable populations (such as older adults, pregnant women, and children) (7) are more likely to be recommended to get the influenza vaccine, so the vaccination uptake and recommendation rate of HCWs may differ among different hospital departments. However, participants from other departments, such as the department of geriatrics, pediatric department, etc., are fewer, which make it difficult to do a more detailed analysis. Furthermore, some HCWs may have misconceptions about influenza and influenza vaccination or are unaware of latest national guidelines and reimbursement policy for the influenza vaccine (12, 29, 41), but we did not take into account this information (such as their knowledge of influenza and influenza vaccination, perception of vaccine effectiveness and side effects, perceived risk of influenza, the vaccine price, etc.,), so there might be confounding factors that were not investigated, which we will explore further in our future study. Additionally, vaccination-related behaviors were reported by the HCWs themselves and, thus, could be biased. Finally, the recommendation efficiency of HCWs who did not track the vaccination status of patients could not be evaluated. Considering that HCWs who track patients' vaccination status may have more awareness of vaccination importance to their patients and spend more efforts in advocating vaccination for them, our study may have overestimated the efficiency of recommendations.

This is the first nationwide, China-based study to investigate the current status of HCWs' engagement with each phase of a vaccination-related-behavior chain, and it would be helpful to obtain a more comprehensive understanding of HCWs' role in fighting vaccine-preventable diseases in China. This is also the first study to explore the impact performing vaccine-focused SDL has on HCWs' engagement in vaccination-related behaviors; thus, an alternative, potentially more efficient intervention could be suggested. We found that the pattern of engagement in vaccination-related behaviors among Chinese HCWs must be improved. It is necessary to develop interventions to help HCWs understand vaccination policy and enhance their awareness of their roles in public vaccination. Importantly, performing vaccine-focused SDL is positively associated with engaging in vaccination-related behaviors and, thus, could be utilized to foster behavior change and ultimately improve vaccination coverage.

## Data availability statement

The raw data supporting the conclusions of this article will be made available by the authors, without undue reservation.

## Ethics statement

The studies involving human participants were reviewed and approved by the Medical Ethics Committee of the Chinese Academy of Medical Sciences and Pecking Union Medical College, Beijing, China. The patients/participants provided their written informed consent to participate in this study.

## Author contributions

LM, LF, and YM designed the study. DL, YY, and YX were responsible for data collection. YM, XH, and WL performed the statistical analysis and drafted the manuscript. YM, WY, LF, and LM engaged in further writing and review. All authors contributed to the article and approved the submitted version.

## Funding

This study was funded by the Non-profit Central Research Institute Fund of Chinese Academy of Medical Sciences (No. 2021-RC330-002), the Discipline Construction Funds of Population Medicine from Peking Union Medical College (No. WH10022021145), and Guilin Talent Mini-highland Scientific Research Project for COVID-19 Prevention and Control [Municipal Committee Talent Office of Guilin City (2020) No. 3-05]. The funding source had no role in the study design, in the collection, analysis, interpretation of data, in the writing of the report, and in the decision to submit the article for publication.

## Conflict of interest

DL was employed by Breath Circles Network Platform. The remaining authors declare that the research was conducted in the absence of any commercial or financial relationships that could be construed as a potential conflict of interest.

## Publisher's note

All claims expressed in this article are solely those of the authors and do not necessarily represent those of their affiliated organizations, or those of the publisher, the editors and the reviewers. Any product that may be evaluated in this article, or claim that may be made by its manufacturer, is not guaranteed or endorsed by the publisher.
