# Supplementary material for: Self-reported vaccination-related behavior patterns among healthcare workers and the association with self-directed learning frequency: A nationwide cross-sectional survey
Source: Front Public Health. 2022 Oct 21;10:951818. doi: 10.3389/fpubh.2022.951818 (PMC9634157; doi:10.3389/fpubh.2022.951818)
Supplement: Supplementary file 1 [file Data_Sheet_1.docx]

Supplementary Material

# Supplementary Tables

**Supplementary Table 1.** Vaccine-related topics for vaccine-focused SDL

| Vaccine-related topics | N (%) | >=1 time/week (N=596) | 1 time/month to <1 time/week (N=634) | 1 time/6 months to <1 time/month (N=340) | <1 time/6 months (N=156) |
| --- | --- | --- | --- | --- | --- |
| Vaccine safety | 1560(90.38) | 524(87.92) | 589(92.90) | 305(89.71) | 142(91.03) |
| Target populations | 1550(89.80) | 529(88.76) | 585(92.27) | 298(87.65) | 138(88.46) |
| Vaccine efficacy | 1438(83.31) | 500(83.89) | 533(84.07) | 280(82.35) | 125(80.13) |
| Vaccine types | 1344(77.87) | 490(82.21) | 504(79.50) | 243(71.47) | 107(68.59) |
| Immunization procedures | 1161(67.27) | 420(70.47) | 438(69.09) | 211(62.06) | 92(58.97) |
| How vaccines function | 1150(66.63) | 445(74.66) | 425(67.03) | 190(55.88) | 90(57.69) |
| Vaccine development | 698(40.44) | 337(56.54) | 228(35.96) | 91(26.76) | 42(26.92) |
| Vaccine price | 642(37.20) | 263(44.13) | 200(31.55) | 119(35.00) | 60(38.46) |
| Production technology | 425(24.62) | 201(33.72) | 145(22.87) | 55(16.18) | 24(15.38) |
| Others | 17(0.98) | 9(1.51) | 3(0.47) | 3(0.88) | 2(1.28) |

**Supplementary Table 2.** Sources for vaccine-focused SDL

| Sources | N (%) | >=1 time/week (N=596) | 1 time/month to <1 time/week (N=634) | 1 time/6 months to <1 time/month (N=340) | <1 time/6 months (N=156) |
| --- | --- | --- | --- | --- | --- |
| Publicity and education efforts in communities and hospitals | 1162(67.32) | 398(66.78) | 454(71.61) | 222(65.29) | 88(56.41) |
| Literature | 971(56.26) | 360(60.40) | 333(52.52) | 189(55.59) | 89(57.05) |
| Wechat | 907(52.55) | 316(53.02) | 344(54.26) | 175(51.47) | 72(46.15) |
| Television, radio, newspapers | 811(46.99) | 344(57.72) | 286(45.11) | 126(37.06) | 55(35.26) |
| Short videos | 294(17.03) | 96(16.11) | 125(19.72) | 52(15.29) | 21(13.46) |
| Websites | 288(16.69) | 91(15.27) | 109(17.19) | 65(19.12) | 23(14.74) |
| Others | 94(5.45) | 23(3.86) | 35(5.52) | 22(6.47) | 14(8.97) |

**Supplementary Table 3.** Types of vaccines that participants have recommended

| Types of vaccines | N (%) |
| --- | --- |
| Influenza vaccines | 1,617(85.60) |
| COVID-19 vaccines | 1,524(80.68) |
| Pneumococcal vaccines | 1,182(62.57) |
| Human Papillomavirus (HPV) vaccines | 815(43.14) |
| Chickenpox vaccines | 624(33.03) |
| Haemophilus influenzae b (Hib) vaccines | 605(32.03) |
| Rotavirus vaccines | 367(19.43) |
| Herpes zoster vaccines | 263(13.92) |

**Supplementary Table 4.** Reasons that participants do not recommend vaccination to others

| Reasons | N (%) |
| --- | --- |
| Inadequate knowledge about vaccines | 90(51.14) |
| Inadequate knowledge about target population for vaccines | 73(41.48) |
| Absence of national or workplace requirements to do so | 49(27.84) |
| Too busy | 36(20.45) |
| Fear of being misinterpreted as having commercial interests | 31(17.61) |
| Concerns about potential disputes | 28(15.91) |
| Fear of adverse reactions | 20(11.36) |
| Do not think it is necessary to get the vaccination | 13(7.39) |
| No confidence in vaccines | 12(6.82) |
| Others | 7(3.98) |

**Supplementary Table 5.** Engagement in vaccination-related behaviors among different groups of participants

|  | Receive vaccines,  N(%) | Recommend routinely, N(%) | Track the recommended, N(%) | Recommend efficiently, N(%) |
| --- | --- | --- | --- | --- |
| Work-related to vaccination |  |  |  |  |
| Yes | 410(51.57) | 527(66.29) | 350(44.03) | 322(40.50) |
| No | 483(38.03) | 522(41.10) | 245(19.29) | 220(17.32) |
| χ2 | 36.53 | 124.10 | 145.83 | 135.72 |
| p | <0.001 | <0.001 | <0.001 | <0.001 |
| Workplace |  |  |  |  |
| Community health center | 77(49.04) | 88(56.05) | 58(36.94) | 55(35.03) |
| Hospital | 733(43.09) | 884(51.97) | 498(29.28) | 450(26.46) |
| χ2 | 2.07 | 0.96 | 4.03 | 5.34 |
| p | 0.150 | 0.327 | 0.045 | 0.021 |
| Department |  |  |  |  |
| Respiratory department | 377(43.63) | 494(57.18) | 255(29.51) | 224(25.93) |
| Non-respiratory department | 356(42.53) | 390(46.59) | 243(29.03) | 226(27.00) |
| χ2 | 0.210 | 19.07 | 0.048 | 0.253 |
| p | 0.646 | <0.001 | 0.827 | 0.615 |
| SDL frequency |  |  |  |  |
| ≥1 time/week | 322(54.03) | 405(67.95) | 305(51.17) | 284(47.65) |
| 1 time/month to <1 time/week | 272(42.90) | 350(55.21) | 189(29.81) | 169(26.66) |
| 1 time/6 months to <1 time/month | 143(42.06) | 155(45.59) | 72(21.18) | 63(18.53) |
| Never to <1 time/6 months | 156(31.52) | 139(28.08) | 29(5.86) | 26(5.25) |
| χ2 | 56.20 | 181.00 | 282.43 | 264.28 |
| p | <0.001 | <0.001 | <0.001 | <0.001 |

**Supplementary Table 6.** Factors associated with vaccination-related behaviors in all participants

|  | Receive vaccination | Recommend vaccination routinely | Track the vaccination status of those recommended to get vaccines | Recommend vaccination efficiently |
| --- | --- | --- | --- | --- |
| **SDL frequency** | | | | |
| Never to <1 time/6 months | Ref | Ref | Ref | Ref |
| 1 time/6 months to <1 time/month | 1.46(1.08-1.98) | 1.60(1.16-2.20) | 2.16(1.48-3.14) | 1.10(0.40-3.00) |
| 1 time/month to <1 time/week | 1.50(1.15-1.96) | 2.30(1.74-3.05) | 3.08(2.20-4.31) | 1.35(0.55-3.29) |
| ≥1 time/week | 2.30(1.74-3.03) | 4.46(3.30-6.04) | 6.18(4.35-8.76) | 1.99(0.79-5.04) |
| **Age group** |  |  |  |  |
| <30 | Ref | Ref | Ref | Ref |
| 30-39 | 1.04(0.69-1.56) | 1.19(0.77-1.83) | 1.06(0.65-1.71) | 0.68(0.22-2.06) |
| 40-49 | 1.07(0.63-1.82) | 1.07(0.61-1.89) | 1.27(0.68-2.37) | 0.52(0.11-2.35) |
| ≥50 | 1.16(0.61-2.21) | 1.03(0.52-2.04) | 0.88(0.42-1.83) | 0.76(0.12-4.88) |
| **Sex** |  |  |  |  |
| Male | Ref | Ref | Ref | Ref |
| Female | 0.97(0.78-1.21) | 1.10(0.87-1.39) | 0.70(0.55-0.90) | 1.08(0.61-1.92) |
| **Years of professional experience** | | | | |
| <5 | Ref | Ref | Ref | Ref |
| 5-9 | 1.04(0.71-1.53) | 1.05(0.70-1.58) | 0.73(0.46-1.16) | 1.29(0.46-3.67) |
| 10-14 | 1.06(0.68-1.66) | 1.35(0.84-2.17) | 0.85(0.50-1.45) | 2.11(0.64-6.92) |
| 15-19 | 0.96(0.56-1.65) | 1.63(0.92-2.88) | 0.62(0.33-1.16) | 3.70(0.75-18.32) |
| ≥20 | 0.96(0.54-1.70) | 2.36(1.27-4.37) | 0.95(0.48-1.88) | 2.77(0.56-13.65) |
| **Educational attainment** | | | | |
| Bachelor’s degree and below | Ref | Ref | Ref | Ref |
| Master’s degree and above | 1.11(0.86-1.44) | 1.29(0.98-1.70) | 0.57(0.43-0.77) | 0.47(0.22-0.99) |
| **Institution** |  |  |  |  |
| Hospital | Ref | Ref | Ref | Ref |
| Community health center | 1.18(0.83-1.69) | 0.95(0.65-1.41) | 1.02(0.68-1.54) | 1.13(0.43-2.99) |
| CDC | 1.98(1.13-3.47) | 1.17(0.64-2.12) | 0.63(0.35-1.16) | 0.98(0.20-4.82) |
| Medical schools or research institutes | 1.04(0.53-2.05) | 0.76(0.36-1.62) | 0.57(0.24-1.33) | 0.82(0.09-7.15) |
| **Occupation** |  |  |  |  |
| Doctor | Ref | Ref | Ref | Ref |
| Nurse | 1.48(1.14-1.92) | 0.48(0.37-0.64) | 1.34(1.00-1.79) | 1.98(0.91-4.29) |
| Technician | 0.93(0.61-1.41) | 0.52(0.34-0.81) | 1.50(0.93-2.40) | 1.96(0.54-7.04) |
| Medical school students or researchers | 0.36(0.19-0.67) | 0.42(0.22-0.82) | 0.59(0.28-1.21) | 4.07(0.59-28.26) |
| Others | 1.15(0.72-1.83) | 0.51(0.31-0.85) | 1.14(0.67-1.96) | 1.35(0.35-5.24) |
| **Job title** |  |  |  |  |
| None | Ref | Ref | Ref | Ref |
| Junior | 0.92(0.59-1.45) | 0.93(0.57-1.50) | 0.56(0.32-0.96) | 2.35(0.85-6.48) |
| Middle | 1.28(0.77-2.10) | 1.03(0.60-1.76) | 0.62(0.34-1.12) | 2.38(0.75-7.61) |
| Senior | 1.48(0.86-2.55) | 1.21(0.67-2.17) | 0.51(0.27-0.97) | 2.76(0.74-10.34) |
| **Performing vaccination-related work** | | | | |
| No | Ref | Ref | Ref | Ref |
| Yes | 1.41(1.15-1.74) | 1.84(1.48-2.30) | 1.43(1.14-1.79) | 0.87(0.50-1.51) |
| **Get the flu vaccine or pneumococcal vaccine** | | | | |
| No | - | Ref | Ref | Ref |
| Yes | - | 2.20(1.79-2.70) | 1.55(1.25-1.93) | 1.69(0.98-2.91) |
| **Recommend vaccination routinely** | | | | |
| No | - | - | Ref | Ref |
| Yes | - |  | 2.43(1.94-3.05) | 1.02(0.57-1.82) |
| **Geographic regions*** | | | | |
| Eastern regions | Ref | Ref | Ref | Ref |
| Central regions | 0.57(0.41-0.80) | 1.58(1.10-2.29) | 0.71(0.48-1.06) | 3.12(1.04-9.39) |
| Western regions | 0.38(0.28-0.52) | 1.73(1.24-2.41) | 0.90(0.63-1.28) | 1.22(0.53-2.81) |

* Regions were divided according to the National Bureau of Statistics of China. Eastern regions include Beijing, Tianjin, Hebei, Liaoning, Shanghai, Jiangsu, Zhejiang, Fujian, Shandong, Guangdong, and Hainan. Central regions include Shanxi, Jilin, Heilongjiang, Anhui, Jiangxi, Henan, Hubei, and Hunan. Western regions include Inner Mongolia, Guangxi, Chongqing, Sichuan, Guizhou, Yunnan, Shaanxi, Gansu, and Qinghai.

**Supplementary Table 7.** Factors associated with vaccination-related behaviors in participants working in hospitals

|  | Receive vaccination | Recommend vaccination routinely | Track the vaccination status of those recommended to get vaccines | Recommend vaccination efficiently |
| --- | --- | --- | --- | --- |
| **SDL frequency** | | | | |
| Never to <1 time/6 months | Ref | Ref | Ref | Ref |
| 1 time/6 months to <1 time/month | 1.44(1.04-1.99) | 1.67(1.19-2.35) | 1.80(1.20-2.69) | 1.01(0.34-3.01) |
| 1 time/month to <1 time/week | 1.38(1.03-1.85) | 2.19(1.62-2.97) | 2.92(2.04-4.18) | 1.27(0.49-3.30) |
| ≥1 time/week | 2.11(1.55-2.88) | 4.80(3.43-6.72) | 6.33(4.33-9.27) | 2.28(0.82-6.35) |
| **Age group** |  |  |  |  |
| <30 | Ref | Ref | Ref | Ref |
| 30-39 | 1.15(0.73-1.82) | 1.02(0.63-1.65) | 0.97(0.57-1.65) | 0.71(0.21-2.35) |
| 40-49 | 1.52(0.84-2.74) | 1.02(0.55-1.91) | 1.24(0.62-2.45) | 0.34(0.07-1.77) |
| >=50 | 1.83(0.90-3.70) | 1.23(0.58-2.62) | 0.74(0.33-1.66) | 0.55(0.07-4.20) |
| **Sex** |  |  |  |  |
| Male | Ref | Ref | Ref | Ref |
| Female | 1.04(0.82-1.33) | 1.09(0.85-1.41) | 0.69(0.53-0.91) | 1.38(0.73-2.60) |
| **Years of professional experience** | | | | |
| <5 | Ref | Ref | Ref | Ref |
| 5-9 | 0.89(0.58-1.39) | 1.10(0.69-1.75) | 0.73(0.44-1.22) | 0.98(0.32-3.06) |
| 10-14 | 1.03(0.62-1.72) | 1.54(0.90-2.63) | 0.93(0.51-1.68) | 2.25(0.59-8.64) |
| 15-19 | 0.79(0.43-1.44) | 1.69(0.89-3.20) | 0.58(0.29-1.17) | 5.46(0.88-33.83) |
| ≥20 | 0.82(0.43-1.58) | 2.42(1.21-4.84) | 1.03(0.49-2.21) | 4.45(0.75-26.34) |
| **Educational attainment** | | | | |
| Bachelor’s degree and below | Ref | Ref | Ref | Ref |
| Master’s degree and above | 1.06(0.80-1.41) | 1.25(0.93-1.69) | 0.57(0.42-0.79) | 0.64(0.29-1.42) |
| **Occupation** |  |  |  |  |
| Doctor | Ref | Ref | Ref | Ref |
| Nurse | 1.34(1.00-1.78) | 0.45(0.33-0.61) | 1.33(0.96-1.85) | 1.82(0.77-4.31) |
| Technician | 1.21(0.76-1.92) | 0.65(0.40-1.07) | 1.43(0.84-2.45) | 0.89(0.23-3.41) |
| Others | 0.64(0.39-1.05) | 0.49(0.29-0.84) | 0.83(0.47-1.49) | 1.40(0.33-5.87) |
| **Department** |  |  |  |  |
| Respiratory | Ref | Ref | Ref | Ref |
| Others | 1.24(1.00-1.54) | 1.88(1.49-2.37) | 1.03(0.80-1.32) | 0.71(0.38-1.33) |
| **Job title** |  |  |  |  |
| None | Ref | Ref | Ref | Ref |
| Junior | 0.93(0.54-1.61) | 0.85(0.48-1.51) | 0.84(0.44-1.59) | 3.01(0.93-9.75) |
| Middle | 1.35(0.73-2.48) | 1.06(0.56-2.01) | 0.93(0.46-1.89) | 3.28(0.78-13.76) |
| Senior | 1.39(0.71-2.71) | 1.07(0.53-2.16) | 0.77(0.36-1.66) | 3.13(0.61-15.95) |
| **Performing vaccination-related work** | | | | |
| No | Ref | Ref | Ref | Ref |
| Yes | 1.33(1.07-1.67) | 1.98(1.55-2.51) | 1.44(1.13-1.84) | 0.80(0.44-1.46) |
| **Working in a hospital that offers on-site vaccination** | | | | |
| No | Ref | Ref | Ref | Ref |
| Yes | 1.92(1.49-2.48) | 1.24(0.95-1.62) | 1.32(0.99-1.76) | 1.82(0.91-3.64) |
| **Get the flu vaccine or pneumococcal vaccine** | | | | |
| No | - | Ref | Ref | Ref |
| Yes | - | 2.12(1.69-2.65) | 1.46(1.16-1.85) | 1.60(0.89-2.88) |
| **Recommend vaccination routinely** | | | | |
| No | - | - | Ref | Ref |
| Yes | - | - | 2.59(2.02-3.32) | 0.77(0.39-1.51) |
| **Geographic regions*** | | | | |
| Eastern regions | Ref | Ref | Ref | Ref |
| Central regions | 0.56(0.38-0.81) | 1.70(1.13-2.56) | 0.65(0.42-1.01) | 2.43(0.74-7.96) |
| Western regions | 0.33(0.23-0.46) | 1.64(1.13-2.38) | 0.76(0.51-1.13) | 0.92(0.36-2.34) |

* Regions were divided according to the National Bureau of Statistics of China. Eastern regions include Beijing, Tianjin, Hebei, Liaoning, Shanghai, Jiangsu, Zhejiang, Fujian, Shandong, Guangdong, and Hainan. Central regions include Shanxi, Jilin, Heilongjiang, Anhui, Jiangxi, Henan, Hubei, and Hunan. Western regions include Inner Mongolia, Guangxi, Chongqing, Sichuan, Guizhou, Yunnan, Shaanxi, Gansu, and Qinghai.
